# Distributed sensing of microseisms and teleseisms with submarine dark fibers

**DOI:** 10.1038/s41467-019-13262-7

**Published:** 2019-12-18

**Authors:** Ethan F. Williams, María R. Fernández-Ruiz, Regina Magalhaes, Roel Vanthillo, Zhongwen Zhan, Miguel González-Herráez, Hugo F. Martins

**Affiliations:** 1 Seismological Laboratory, California Institute of Technology, 1200 E. California Boulevard, Pasadena, CA 91125-2100 USA; 2 Department of Electronics, University of Alcalá, Polytechnic School, 28805 Alcalá de Henares, Spain; 3 Marlinks, Sint-Maartenstraat 5, 3000 Leuven, Belgium; 4 Instituto de Óptica, CSIC, 28006 Madrid, Spain

**Keywords:** Ocean sciences, Solid Earth sciences, Optics and photonics

## Abstract

Sparse seismic instrumentation in the oceans limits our understanding of deep Earth dynamics and submarine earthquakes. Distributed acoustic sensing (DAS), an emerging technology that converts optical fiber to seismic sensors, allows us to leverage pre-existing submarine telecommunication cables for seismic monitoring. Here we report observations of microseism, local surface gravity waves, and a teleseismic earthquake along a 4192-sensor ocean-bottom DAS array offshore Belgium. We observe in-situ how opposing groups of ocean surface gravity waves generate double-frequency seismic Scholte waves, as described by the Longuet-Higgins theory of microseism generation. We also extract P- and S-wave phases from the 2018-08-19 ${M}_{w}8.2$ Fiji deep earthquake in the 0.01-1 Hz frequency band, though waveform fidelity is low at high frequencies. These results suggest significant potential of DAS in next-generation submarine seismic networks.

## Abstract (web summary)

Distributed acoustic sensing (DAS) technology in geophysics is commonly known for applications such as active source seismic profiling in boreholes. Here, the authors convert the fiber optics cable into an ocean bottom seismic recording array with thousands of single component channels.

## Introduction

One of the greatest outstanding challenges in seismology is the sparsity of instrumentation across Earth’s oceans^1,2^. Poor spatial coverage results in biases and low-resolution regions in global tomography models as well as significant location uncertainty for offshore seismicity. Modern ocean-bottom seismometers (OBS) generally fall into two categories: short-period instruments ($\sim$1–5 Hz), which can record for up to a month or more, and long-period or broadband instruments (BBOBS), which often employ the same sensors as terrestrial broadband seismic stations and can operate for as long as 2 years^3^. Whereas short-period instruments are primarily used in active-source experiments, BBOBS are ideal for passive-source experiments and have been used for tomographic studies, earthquake location, and ocean wave monitoring among numerous other applications^4–12^. However, BBOBS are expensive and limited by data telemetry and battery life except in near-shore environments^3^. Recent work has explored several alternatives to conventional BBOBS for offshore seismic monitoring, including free-floating robots equipped with hydrophones^13^, moored surface buoys or autonomous surface vehicles for satellite telemetry acoustically linked to BBOBS^14,15^, and cabled arrays of broadband sensors^16^. Recently, Marra et al.^17^ applied laser interferometry to convert long ocean-bottom telecommunications optical fiber links into seismic strainmeters. This work is particularly promising because repurposing the  >1 million km of pre-existing trans-oceanic telecommunications cables as seismic sensors would permit rapid detection and location of earthquakes throughout the world’s ocean basins. Unfortunately, the particular technique in Marra et al.^17^ is limited to measuring propagation delays integrated across an entire cable length, resulting in a single seismograph with equivalent station location uncertainty on the order of 1 km and complicated instrument response.

Distributed acoustic sensing (DAS) is an emerging technology with strong potential to form the core of next-generation submarine seismic monitoring infrastructure. A DAS interrogator unit probes a fiber optic cable with a coherent laser pulse and measures changes in the phase of the returning optical backscatter time-series. Optical phase shifts between pulses are proportional to longitudinal strain in the fiber and can be mapped into the finite, distributed strain across a fiber segment (termed gauge length) by integration. Applying DAS technology to a fiber optic cable effectively converts the cable into a seismic recording array with thousands of single-component channels, real-time data telemetry, and unlimited deployment duration as long as the DAS unit is powered. For about a decade, DAS has been successfully utilized in boreholes for active-source seismic profiling^18–20^. Recent work with onshore trenched or conduit-installed horizontal fibers has demonstrated the ability of DAS arrays to record earthquakes and other seismic signals at local to teleseismic distances with high waveform fidelity^21–28^.

In this paper, we demonstrate that submarine horizontal DAS arrays utilizing pre-existing ocean-bottom fiber optic cables are similarly effective for seismological studies and can also record pressure perturbations from ocean wave phenomena. We first examine ocean surface gravity waves and associated seismic modes directly observed on an ocean-bottom DAS array offshore Zeebrugge, Belgium, which we interpret as evidence of in situ microseism generation. We then report our observation of body waves from the 2018-08-19 ${M}_{w}8.2$ Fiji deep earthquake. Finally, we discuss implications for future DAS deployments in marine settings.

## Results

### Experiment overview

**Fig. 1:**
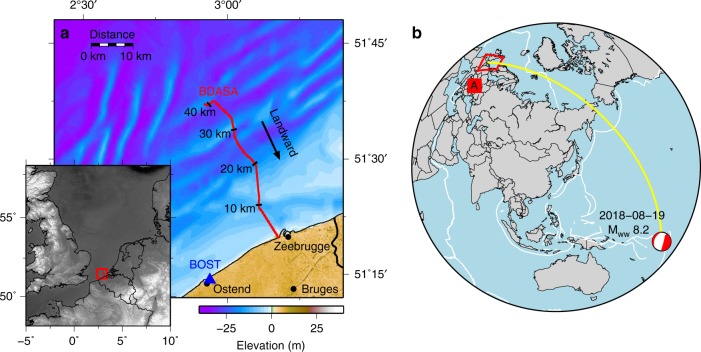
Array location. **a** Local map showing the location Belgium Distributed Acoustic Sensing Array (BDASA, red line) and nearby broadband station BOST (blue triangle), with a regional map inset. **b** World map showing the location of the array (red box), the GCMT solution for the 2018-08-19 *M*_*w*_8.2 Fiji deep earthquake, and great circle path between the earthquake epicenter and the array (yellow).

The Belgium DAS array (BDASA) occupied a pre-existing ocean-bottom fiber optic cable in the Southern Bight of the North Sea offshore Zeebrugge, Belgium (Fig. 1). During August of 2018, the BDASA recorded continuously for nearly a month. Here we analyze the 1-h record containing the principal body wave phases from the 2018-08-19 ${M}_{w}8.2$ Fiji deep earthquake, along with ocean wave signals and microseism noise. The fiber optic cable was originally installed to monitor a power cable for the Belwind Offshore Wind Farm (cable and fiber specifications are given in the Supplementary Note 1, Supplementary Fig. 1). Cable geometry is approximately straight over four 10-km segments and is flat or shallowly dipping, except for a steep channel around 10 km and two $\sim$15-m bathymetric ridges at $\sim$30 and 40 km from the coast (Fig. 1a). The cable is buried between 0.5 and 3.5 m below the seafloor in water depths shallower than 40 m. A chirped-pulse DAS system built and installed by the University of Alcala^29^ continuously interrogated a 42-km near-shore segment of the fiber with channel spacing of 10 m, creating 4192 simultaneously recording seismic sensors (see “Methods”).

In “Separation of coherent signals,” we first decompose the raw BDASA data in the frequency–wavenumber domain, separating and identifying oceanic and seismic signals. In “Microseism generation,” we compare our observations of ocean surface gravity and Scholte waves to the Longuet-Higgins^30^ theory of double-frequency microseism generation. In “Ocean waves and ocean currents,” we describe sea state and ocean currents across the BDASA, evident from variations in the symmetry of ocean surface gravity wave dispersion. Finally, we discuss the quality of teleseismic body waves from 2018-08-19 ${M}_{w}8.2$ Fiji deep earthquake, recovered from the BDASA after filtering out ocean wave and microseism signals.

### Separation of coherent signals

**Fig. 2:**
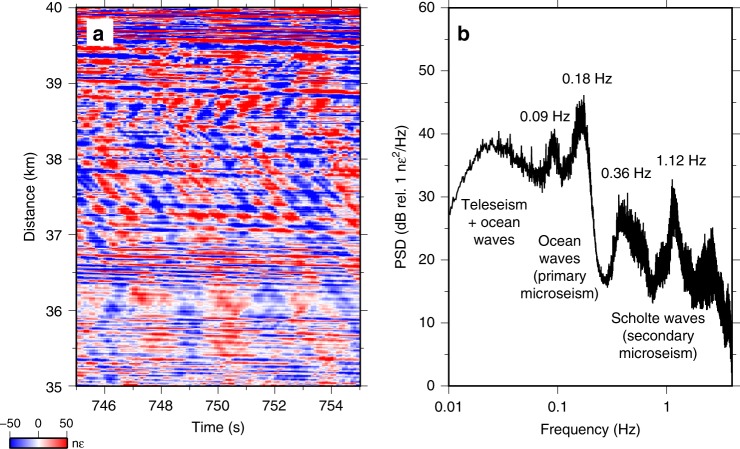
Raw DAS data. **a** Ten seconds of raw distributed acoustic sensing (DAS) data along the last 5 km of the array illustrating the superposition of coherent signals from ocean and seismic waves propagating both landward and seaward across the array. **b** Mean power spectral density (PSD) of raw DAS strain data over the complete 1-h record between 35 and 40 km (same position as **a**).

**Fig. 3:**
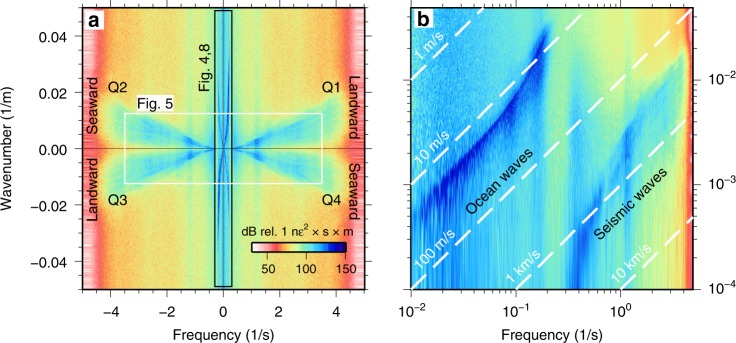
Separation of ocean and seismic waves. **a** Raw frequency–wavenumber power spectrum of 1 h of strain data across the full 42-km array. **b** Quadrant 1 (landward-propagating waves) plotted in logarithmic space, showing coherent ocean wave energy at low frequencies and coherent seismic wave energy at high frequencies. Dashed white lines are plotted along contours of constant phase velocity ($c=f/k$).

In the time domain, raw strain records from the BDASA are complicated by the superposition of several coherent signals with incoherent noise from sources such as temperature drift (Fig. 2a). In the frequency domain, the power spectral density (PSD) of each channel exhibits five distinct peaks, corresponding to different wave modes propagating across the array (Fig. 2b). In order to identify and interpret the wave types comprising each peak, we apply a two-dimensional (2D) Fast Fourier Transform from the raw strain records into the frequency–wavenumber (*f*-*k*) domain (Fig. 3). *F*-*k* domain analysis of the raw BDASA data is possible here because the chirped-pulse DAS system exhibits negligible fading of sensitivity along the fiber, as is common in conventional DAS and that would require pre-processing at the expense of bandwidth (see “Methods”). Given the quasi-linear geometry of the fiber cable, no corrective algorithms or fiber sectioning methods were applied to compensate cable turns, resulting in slight smearing of energy along the wavenumber axis.

Visualization of BDASA data in the *f*-*k* domain allows identification and separation of coherent seismic and oceanic signals in each frequency band based on their characteristic phase velocities ($c=f/k$). Figure 3a shows the complete 4192-channel, 1-h dataset transformed into a single *f*-*k* spectrum. Energy in quadrants 1 and 3 corresponds to waves with positive phase velocities. In the coordinate system we adopted, this represents waves propagating landward across the array. Similarly, energy in quadrants 2 and 4 corresponds to waves with negative phase velocities, propagating seaward across the array. There are two distinct groups of energy in the *f*-*k* spectrum, which are easily visualized in log–log space (Fig. 3b). Ocean waves appear at low frequencies (<0.3 Hz) with apparent phase velocity slower than $\sim$17 m/s. Seismic waves appear at high frequencies (>0.3 Hz) with apparent phase velocity faster than $\sim$300 m/s. Teleseismic body waves from the ${M}_{w}8.2$ Fiji deep earthquake are not directly visible in the *f*-*k* spectrum.

#### Ocean surface gravity waves

**Fig. 4:**
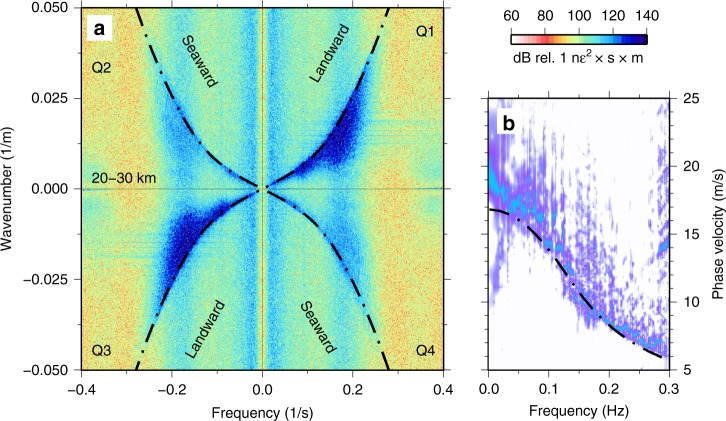
Ocean surface gravity waves. **a** Raw distributed acoustic sensing frequency–wavenumber (*f*-*k*) spectrum calculated over 10 min between 20 and 30 km, showing strong landward-propagating and weak seaward-propagating ocean surface gravity waves. **b** The *f*-*k* spectrum from quadrant 1 of **a** projected into phase velocity space showing coherent dispersion from $\sim {\!}17$ m/s at small wavenumbers to $\sim {\!}6$ m/s at 0.3 Hz (each frequency bin is normalized). Both **a** and **b** are overlaid with the theoretical dispersion curve for ocean surface gravity waves, evaluated at a water depth of 25 m (black).

Surface gravity and infragravity waves are excited in oceanic waters by wind–sea interaction. Ocean surface gravity waves follow the dispersion relation ${\omega }^{2}=gk\,\text{tanh}\,(kH)$, where $\omega$ is angular frequency, $g$ is gravitational acceleration, $k$ is angular wavenumber, and $H$ is water depth (e.g., ref. ^31^). *F*-*k* analysis of BDASA data shows strong, coherent energy packets in all four quadrants between <0.01 and 0.3 Hz (Fig 4a) with peaks at 0.09 and 0.18 Hz (Fig. 2b). The upper edge of these packets follows the ocean surface gravity wave dispersion relation, corresponding to energy propagating axially along the cable both landward and seaward. Energy appearing below this edge represents surface gravity waves with faster apparent phase velocity that obey the same dispersion relation but are obliquely incident to the cable. For the 20–30-km cable segment shown in Fig. 4a, landward-propagating ocean surface gravity waves are stronger than seaward-propagating waves.

We project the *f*-*k* spectrum into frequency–phase velocity space (*f*-*c*) using the coordinate transformation $c=f/k$, permitting better visualization of phase velocity dispersion (Fig. 4b). In *f*-*c* space, ocean surface gravity waves exhibit coherent dispersion from faster phase velocity ($\sim {\!}17$ m/s) at low frequencies ($\sim {\!}0.01$ Hz) to slower phase velocity ($\sim {\!}6$ m/s) at 0.3 Hz. Ocean wave energy tapers off quickly above 0.3 Hz.

#### Scholte (seismic) waves

**Fig. 5:**
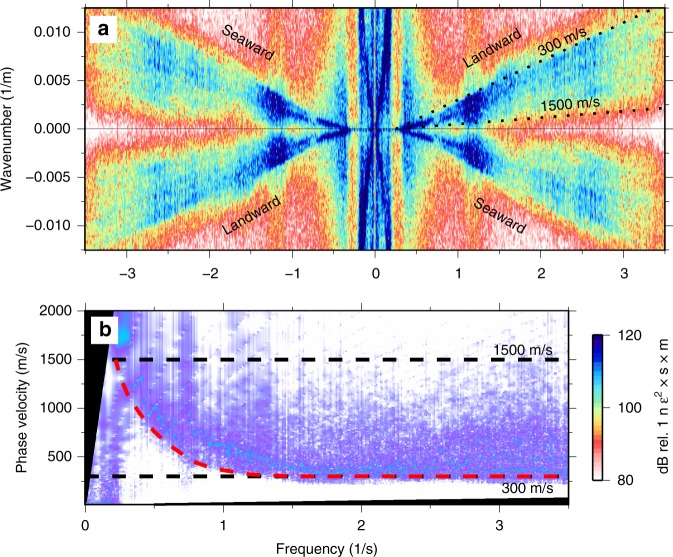
Scholte (seismic) waves. **a** Raw distributed acoustic sensing frequency–wavenumber (*f*-*k*) spectrum calculated over 1 h between 35 and 40 km, showing symmetric landward- and seaward-propagating Scholte waves between 0.3 and 3.5 Hz. **b** The *f*-*k* spectrum from quadrant 1 of **a** projected into phase velocity space showing coherent dispersion from $\sim {\!}1500$ m/s at 0.36 Hz to $\sim {\!}300$ m/s above 1 Hz (each frequency bin is normalized). Both **a** and **b** are overlaid with contours of constant velocity at 1500 and 300 m/s (black), and an approximate dispersion curve is hand-drawn in **b** (red).

Seismic waves propagating faster than 300 m/s are represented in the *f*-*k* domain by symmetric fans of energy at frequencies  >0.3 Hz (Fig. 5a) with peaks at 0.36 and 1.12 Hz (Fig. 2b). When projected from the *f*-*k* domain into *f*-*c* space, the high-frequency energy packet exhibits strong dispersion from phase velocities close to the compressional velocity of water ($\sim$1500 m/s) at 0.36 Hz to an asymptotic velocity of $\sim$300 m/s above 1 Hz (Fig. 5b). This is consistent with the expected dispersion relation of Scholte waves along the sediment–water interface, which follows the compressional velocity of water at low frequencies and the shear-wave velocity of the shallow sediment layer at high frequencies^32^. As for ocean waves, the low-velocity edge of the *f*-*k* energy packets in each quadrant represents Scholte waves propagating axially along the cable. Energy appearing at faster apparent phase velocities represents Scholte waves obliquely incident to the cable. We note that the 0.3–3.5 Hz Scholte waves are observed in the 550 s of data preceding the arrival of the first P-wave phases from the Fiji earthquake and therefore must be an independent, local phenomenon.

### Microseism generation

Globally, seismograms record broadband seismic noise with peaks around 14- and 7-s period, termed microseisms, which have long been attributed to ocean wave sources (e.g., ref. ^33^). The longer period (lower frequency) peak is commonly referred to as primary microseism, while the shorter period (higher frequency) peak is called secondary microseism. Source locations of primary microseism appear to be restricted to coastal areas, with seismic noise excited by direct loading of the seafloor where gravity waves impinge on shallow coastal waters^34,35^. Source locations of secondary microseism, however, include both near-shore and deep-water environments^35,36^, and the amplitude of the secondary microseism peak has not been tied directly to coastal ocean wave conditions (e.g., ref. ^37^). While the relative amplitude and central frequencies of the microseism peaks vary by region and sea state, the double-frequency relationship between primary and secondary microseism is universal and a subject of continued research. Here we argue that ocean surface gravity waves and Scholte waves observed on the BDASA at double-frequency (0.18 and 0.36 Hz, respectively) together represent in situ microseism generation following the theory of Longuet-Higgins^30^.

#### Primary microseism and its depth dependence

Based on our *f*-*k* analysis above, the 0.18-Hz peak in Fig. 2b corresponds to ocean surface gravity waves propagating across the BDASA. Because the cable is buried at a depth of 0.5–3.5 m, the BDASA is only mechanically coupled to the water body above through the intermediary shallow sediment layer, so ocean waves cannot be observed directly. Instead, ocean waves signals observed on the BDASA are poroelastic strains in the solid earth induced by the pressure field of ocean waves propagating above, hence primary microseism generated in situ by ocean wave loading. Common observations of primary microseism on terrestrial seismic networks (e.g., ref. ^35^) constitute diffuse seismic energy radiated into the far field, whereas here we observe the primary microseim source directly.

**Fig. 6:**
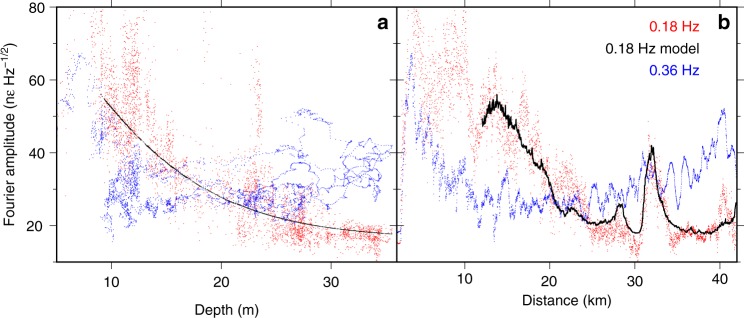
Depth and distance scaling. **a** Fourier components of the raw distributed acoustic sensing strain spectrum at 0.18 (primary microseism, red) and 0.36 Hz (secondary microseism, blue) calculated at each channel plotted versus water depth. Also shown is the model of 0.18-Hz noise as a function of theoretical seafloor pressure described in the text (black). **b** Same as **a** but plotted with distance along the fiber.

To test this interpretation, we compare the variation in amplitude of the 0.18-Hz peak to the expected seafloor pressure under ocean surface gravity waves along the cable depth profile. The strength of ocean surface gravity waves decays rapidly with depth, which is why source regions of primary microseism are constrained to the coast. Invoking linear wave theory, the magnitude of the pressure perturbations at the seafloor beneath a surface gravity wave scales with angular wavenumber $k$ and water depth $H$ as ${p}_{d}\propto \,\text{sech}\,(kH)$ (e.g., ref. ^31^). To evaluate ${p}_{d}$, we iteratively solve the implicit dispersion relation for ocean surface gravity waves, ${\omega }^{2}=gk\,\text{tanh}\,(kH)$, to obtain $\omega (k)$, and then calculate a theoretical ${p}_{d}$ as a function of distance and depth using the cable profile. In order to determine a scaling factor between seafloor pressure and fiber strain, we fit the Fourier amplitude observed on the BDASA at 0.18 Hz as a linear function of theoretical ${p}_{d}$ (see Supplementary Note 2), to produce the model plotted in Fig. 6. We observe a good correspondence between the observed and modeled Fourier amplitude at 0.18 Hz with both water depth and distance along the cable (Fig. 6). To leading order, then 0.18-Hz energy observed on the BDASA is proportional to pressure applied by ocean surface gravity waves at the seafloor, confirming our interpretation of primary microseism generation.

#### Secondary microseism by ocean wave interaction

Longuet-Higgins^30^ first proposed a mechanism for the double-frequency nature of microseisms, whereby nonlinear interaction of opposing groups of surface gravity waves at one frequency generates a depth-invariant pressure term of second-order magnitude that oscillates at twice the frequency of the surface waves. Hasselmann^38^ expanded this theory to demonstrate that appreciable microseisms are excited only by components of the ocean pressure field that match the phase velocities of the seismic modes of the coupled water–seabed system. In the simplest case, the phase velocity of Longuet-Higgins’s second-order pressure term scales as $c=2\omega /\parallel {\overrightarrow{k}}_{1}+{\overrightarrow{k}}_{2}\parallel$ for two plane surface gravity waves with phase ${\overrightarrow{k}}_{1}\cdot \overrightarrow{x}-\omega t$ and ${\overrightarrow{k}}_{2}\cdot \overrightarrow{x}-\omega t$. Hence, for opposing waves (when ${\overrightarrow{k}}_{1}$ is close to $-{\overrightarrow{k}}_{2}$), $c$ approaches seismic velocities.

Based on these theories, we assert that the 0.36-Hz Scholte waves discussed above represent secondary microseism associated with the 0.18-Hz opposing surface gravity wave groups. Unlike the 0.18-Hz energy peak, the 0.36-Hz peak observed in the BDASA PSD is almost invariant with depth and is not adequately described by the pressure–depth scaling of ocean surface gravity waves (Fig. 6a). Instead, the Fourier amplitude at 0.36 Hz decreases over the first 12–15 km of the array and then increases gradually with distance out to 40 km (Fig. 6b). Therefore, Scholte waves at 0.36 Hz cannot be the product of direct loading by ocean surface gravity waves.

**Fig. 7:**
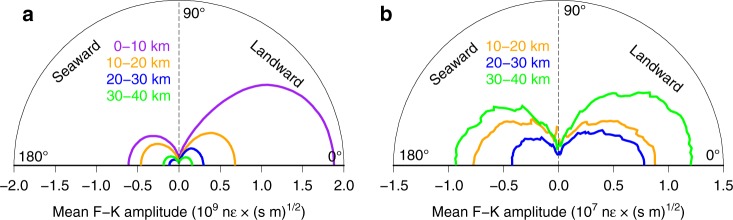
Directional spectra. **a** Mean frequency–wavenumber (*f*-*k*) amplitude of ocean waves (primary microseism) as a function of azimuth calculated between 0.05 and 0.25 Hz using the ocean surface gravity wave dispersion relation for each of the four 10-km array segments. **b** Mean *f*-*k* amplitude of Scholte waves (secondary microseism) as a function of azimuth calculated between 1.5 and 3.5 Hz assuming a true phase velocity of 300 m/s and no dispersion across this frequency band.

Longuet-Higgins^30^ predicts that the amplitude of the secondary pressure term generated by non-linear wave interaction is proportional to the product of the amplitudes of the two opposing ocean wavefield components. Hence, we expect to observe the strongest Scholte waves where seaward- and landward-propagating ocean surface gravity waves are of similar strength and the weakest Scholte waves where seaward- and landward-propagating ocean waves are of significantly different strengths. To test this property, we plot directional spectra for both ocean surface gravity waves and Scholte waves (Fig. 7). For each wave type, theoretical dispersion curves are constructed for waves with different incident azimuths. For each of the four 10-km quasi-linear segments along the fiber, we then take the mean *f*-*k* spectral amplitude interpolated along each dispersion curve to form the polar plots in Fig. 7 (see Supplementary Note 3). The cable segment in water depths $\ < 10$ m is neglected in this analysis, as the PSD of this region is saturated by incoherent energy across a broad band, likely associated with shoaling of ocean waves.

The relative strength of seaward- and landward-propagating ocean surface gravity wavefield components is most similar for the 30–40-km segment, slightly less equal for the 10–20-km segment, and most disparate for the 20–30-km segment (Fig. 7a). As predicted by this scaling, the absolute strength of the Scholte wavefield components (in both quadrants) is greatest for the 30–40-km segment, less for the 10–20-km segment, and smallest for the 20–30-km segment (Fig. 7b). Note that, because Longuet-Higgins’s second-order pressure term does not decay with depth, this result is dependent only on the relative strengths of ocean wavefield components shown in Fig. 7a and not on their absolute strength.

**Fig. 8:**
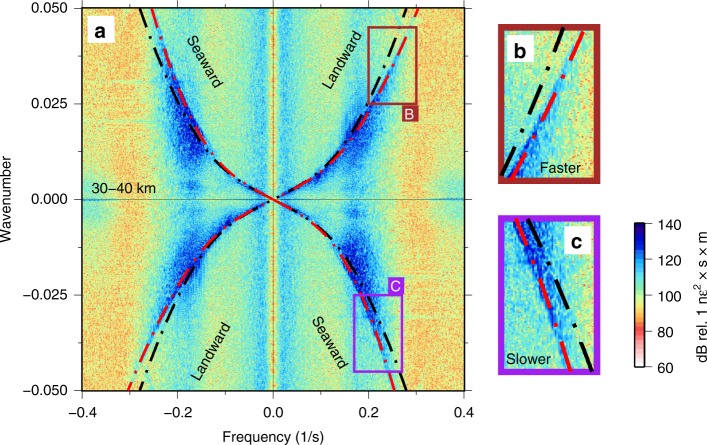
Ocean currents. **a** Raw distributed acoustic sensing frequency–wavenumber spectrum calculated over 10 min between 30 and 40 km, showing asymmetrical dispersion due to an ocean current. **b**, **c** Insets to **a** illustrating how landward-propagating ocean waves exhibit faster velocities than seaward-propagating ocean waves. The theoretical dispersion curves for ocean surface gravity waves are plotted with (red) and without (black) the effect of a mean flow current.

For Scholte (similar to Rayleigh) waves, the theoretical azimuthal sensitivity of DAS is approximately ${\mathrm{cos}}^{2}(\theta )$, where $\theta =0$ is along the axis of the fiber, in the limit that the wavelength is much longer than the gauge length used by the DAS system^39^. The directional spectra shown in Fig. 8b all approximately follow a $co{s}^{2}$ shape, suggesting that the azimuthal distribution of Scholte wave energy is relatively diffuse (or isotropically propagating) along most of the fiber. The diffuse nature of the secondary microseism wavefield is further evidence that these waves must be generated in situ and also offers a direct observation of the radiation pattern of secondary microseism at its source.

Within this framework, we are unable to describe the 1.12-Hz peak (Fig. 2b) and associated high-frequency Scholte wave energy observed up to 3.5 Hz (Fig. 5a). The 1.12-Hz peak likely does not represent secondary microseism associated with a pair of opposing surface gravity wave groups with dominant frequency of 0.55 Hz, as no 0.55-Hz peak is observed in our data. However, the strength of ocean waves observed at the seafloor attenuates strongly with decreasing wavelength, so it is possible that 0.55-Hz ocean waves do exist. The 1.12-Hz peak could also correspond to external environmental noise from an unknown (potentially anthropogenic) source. Alternatively, it could represent a resonant mode of the coupled sediment–water system.

### Ocean waves and ocean currents

Beyond their implications for microseism generation, ocean surface gravity waves observed on the BDASA demonstrate the potential of ocean-bottom DAS for investigations in physical oceanography. Computing *f*-*k* spectra across different segments of the cable, we can distinguish spatial variations in the intensity of landward-propagating versus seaward-propagating ocean surface gravity waves in order to interpret sea state. For example, on the 20–30-km segment (Fig. 4a) landward-propagating waves are stronger than the seaward-propagating waves, while on the 30–40-km segment (Fig. 8a) landward- and seaward-propagating waves are of similar strength (see also Fig. 7a). Because the strength of seaward-propagating waves is greater on the outermost segment of the cable than on the next segment closer to shore, we infer that some of the seaward-propagating waves must be local reflections from the bathymetric ridge at 30 km. Inboard of the 30-km ridge, we observe that the ratio of seaward- to landward-propagating wave energy decreases systematically, which is consistent with the expectation that all seaward-propagating ocean waves observed on the BDASA are generated by reflection from the sloping seabed approaching the coast. While the extent of our interpretation is limited by the 1-h record length of BDASA data, the framework for ocean wave analysis demonstrated here would be easily applicable to monitor temporal variations in sea state over tidal to annual scales.

Because of the large number of channels and high-sample rate on the BDASA, *f*-*k* domain resolution is sufficiently fine to distinguish small perturbations in surface gravity wave dispersion associated with ocean currents. For example, the *f*-*k* spectrum of the last 10-km segment (30–40 km) is asymmetrical and evolves over the 1-h record (only the last 10-min window is shown in Fig. 8). On this segment, landward-propagating waves appear faster than seaward-propagating waves, as the result of an ocean current with a component of flow in the landward direction along the array (Fig. 8b, c). We fit the dispersion asymmetry with a mean flow correction to the dispersion relation ${(\omega -Uk)}^{2}=gk\,\text{tanh}\,(kH)$, which describes the first-order effect of surface gravity waves propagating in a current, where $U$ is the apparent velocity of the current along the cable (as above, $\omega$ is angular frequency, $k$ is angular wavenumber, $g$ is gravitational acceleration, and $H$ is water depth). Over the 1-h record, the strength of the observed current increases gradually from 0.1 to 0.5 m/s apparent velocity in the landward direction. Contemporary methods of ocean current measurement are largely limited to either high-frequency radio observation of surface currents^40,41^ or in situ observation of current depth profiles using spatially sparse moorings, drifters, or ship-board instruments^42–44^. Our observation of spatio-temporal variations in current speed is significant because it suggests potential application of ocean-bottom DAS to in situ measurement and monitoring of ocean currents by exploiting models of wave interaction with heterogeneous currents (e.g., ref. ^45^) to recover high-resolution spatial variations in current speed along an array.

### 2018-08-19 ${M}_{w}8.2$ Fiji deep earthquake

Rapid, accurate measurement of body wave travel times is an essential goal of next-generation broadband marine seismology^1^ and has motivated many recent advances in ocean-bottom seismic instrumentation (e.g., ref. ^13^). Ocean-bottom DAS arrays are an ideal technological solution because they offer real-time telemetry and are intrinsically synchronized (all channels are interrogated with the same unit, thus avoiding any differential clock drift across the array), neither of which are easily achievable features of OBS networks. Northern Europe is a seismically quiescent area, so no local or regional seismic events were recorded. However, the BDASA captured teleseismic body waves from a ${M}_{w}8.2$ deep earthquake in the Fiji-Tonga area on August 19, 2018 (Fig. 1b). Teleseisms arrived from an epicentral distance of 146.7° (>16,300 km), at a back azimuth of 358.5° (27.6° oblique to the mean fiber azimuth of 330.9°). Because the 2018-08-19 Fiji event occurred at a depth of 600 km, only weak surface waves were excited and hence could not be analyzed.

**Fig. 9:**
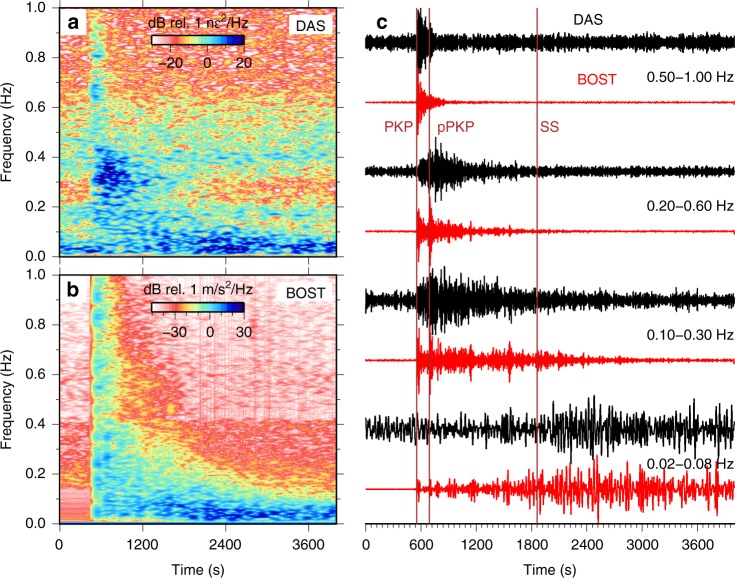
Teleseismic waveforms. **a** Spectrogram of power spectral density (PSD) over time for the *f*-*k* filtered and stacked distributed acoustic sensing (DAS) beam trace (black in **c**), showing strong energy between 0 and 1 Hz around the arrival of the PKP phase around 550 s and <0.1 Hz following the arrival of the SS phase around 1860 s. **b** Spectrogram for the rotated BOST channel (red in **c**), showing the same major features. **c** Stacked DAS beam trace (black) filtered to various bands between 0.02 and 1 Hz compared with amplitude-normalized particle velocity from broadband station BOST rotated into the mean azimuth of the DAS array (red).

Teleseismic body waves from the Fiji earthquake are close to vertically incident and expected to arrive almost simultaneously along the array, hence appearing at wavenumbers lower than can be resolved across a few kilometers aperture. In order to isolate teleseisms from ocean surface gravity and Scholte waves, we apply a 2D band-pass filter in the *f*-*k* domain between 0.001 and 1 Hz and between 0 and 0.002 ${\text{m}}^{-1}$ in the first and third quadrants (corresponding to energy propagating landward across the array from the north/west; Supplementary Fig. 2), stack waveforms across a 5-km array segment to form a beam trace, and finally apply a range of bandpass filters to the beam trace to produce the BDASA waveforms shown in Fig. 9 (see Supplementary Note 4). We compare the BDASA beam trace to nearby broadband seismometer BOST (30–50 km south of BDASA), after rotating the horizontal channels into the mean azimuth of the BDASA and bandpass filtering.

At high frequencies (>0.1 Hz), we recover the PKP phase ($\sim$550 s) and its associated pPKP + sPKP depth phases ($\sim$690 s), the travel times of which correspond well to those recorded on BOST (Fig. 9). The envelopes of the recovered P-phases (not shown) are similar to those from BOST, although the they show low-to-moderate waveform fidelity (mean correlation coefficient of 0.25; Supplementary Fig. 3, Supplementary Note 4). Hence, the polarity of the first P-wave arrival recovered from the BDASA is not reliable across parts of the array. Spatially variable P-waveforms may be physical, however, as high frequency waves can be strongly affected by near-surface structures and the water layer. At low frequencies (<0.15 Hz), the background noise is substantially stronger, but we still recover a complex S-wavetrain, which exhibits moderate-to-high waveform fidelity when compared with BOST (mean correlation coefficient of 0.6; Supplementary Fig. 4). Recovered P- and S-waveforms are both coherent along the length of the array (Supplementary Fig. 5).

Because the BDASA measures strain across a 10-m gauge length, whereas BOST measures particle velocity at a single point, theoretical amplitudes are approximately proportional by a factor of the apparent horizontal slowness for wavelengths longer than twice the gauge length^26^. For the Fiji earthquake, the ratio of BDASA strain amplitude to BOST particle velocity amplitude does not yield reasonable apparent velocities for the observed phases across any band. Hence, we infer that strain-transfer coupling between the solid earth and the BDASA fiber, a consequence of the fiber casing and installation, is complex (see Supplementary Note 1, Supplementary Fig. 1).

While a ${M}_{w}8.2$ deep earthquake is a rare and particularly large event, body wave energy observed in Belgium at 146.7° epicentral distance is lower in spectral amplitude than would be expected for regional earthquakes (<1° epicentral distance) greater than $\sim {\!}M3.5$ (see Supplementary Note 6; Supplementary Fig. 6). Hence, BDASA clearly exhibits teleseismic and regional seismic monitoring capability, as both P-wave and S-wave travel times can be recovered across a broad band, and S-wave polarity is robust over the frequencies of interest to global seismology.

## Discussion

We have presented and analyzed our observations of seismic and ocean waves on an ocean-bottom DAS array offshore Belgium, demonstrating that DAS arrays utilizing existing ocean-bottom fiber optic installations can offer high-value seismographic and oceanographic data products. In particular, we recovered both P- and S-phases from the 2018-08-19 Fiji deep earthquake, though only S-waves exhibited moderate-to-high waveform fidelity. While we were unable to recover robust polarity of high-frequency P-phases, we can expect that ocean-bottom DAS arrays in deep water would have much lower detection thresholds for seismic signals than observed here, as has been demonstrated for OBS (e.g., ref. ^46^). For an ocean-bottom DAS array, the noise floor can be considered as the superposition of instrumental noise from the DAS interrogator unit and fiber, temperature noise from variations in pore fluid temperature, pressure noise from ocean waves, and seismic noise. The aggressive filtering procedure we applied to recover teleseismic waveforms was necessitated to remove environmental signal, not instrument noise, as coherent signals of physical origin were observed across the full band of interest (0.01–5 Hz). Onshore studies with DAS arrays have found that instrument noise is approximately inversely proportional to frequency with a noise floor no higher than 1 $\upmu \upvarepsilon$/Hz${}^{1/2}$ at 1 Hz^47^. Laboratory experiments show that, in a stable temperature environment, DAS systems can exhibit a noise floor <100 p$\upvarepsilon$/Hz${}^{1/2}$ at 1 Hz^48^. On a DAS array, a temperature perturbation of 1 mK is indistinguishable from a 10-n$\upvarepsilon$ strain, so high-frequency temperature fluctuations along the fiber can contribute spurious signals. Water-bottom temperatures may vary on the order of 1 K at tidal periods in the near-shore environment; however, such variability attenuates strongly with depth and is inversely correlated to frequency (e.g., refs. ^49,50^). Consequently, instrumental and temperature noise are not limiting factors for most seismological applications, as seen here. In deep water settings, the magnitude of pressure oscillations beneath ocean surface gravity waves, the primary environmental noise that dominates BDASA data between 0.01 and 0.26 Hz, decays exponentially with depth. Therefore, the shallow-water setting of the BDASA actually represents a “worst case” environment for recording teleseismic events^46,51^, and thus our ability to recover both P- and S-phase is particularly significant.

Compared to traditional OBS deployments, another advantage of DAS is the number and density of stations. Utilizing hundreds of stations from any segment of the array, we were able to apply array-based processing in order to distinguish seismic and ocean signals based on their phase information. So-called “large N” deployments permit low detection thresholds for small earthquakes, precise location of earthquakes, low uncertainty in travel time measurements, and high-resolution imaging studies^25,52,53^. Further, we have demonstrated that large-N ocean-bottom networks open up new possibilities in studying ocean wave phenomena and microseism generation. The vast majority of studies examining the physics of ocean microseism generation have been limited to remote observation of radiated energy on terrestrial broadband networks^33,37,54,55^. The few studies utilizing ocean-bottom instrumentation to correlate ocean-wave phenomena with microseism in situ have been restricted by small network size, effectively resulting in measurements of microseism direction and intensity at a single point with or without simultaneous ocean wave information, and have had mixed success in validating theoretical models^36,56–60^. Simultaneous observation of ocean pressure variations and seismic noise across several thousand channels on ocean-bottom DAS arrays of arbitrary geometry permits reconstruction of the full surface gravity wave and Scholte wave fields, as shown here, and, with the addition of a time-lapse component to future surveys, offers a leap forward in our ability to study microseism and its source processes.

However, several technological challenges still remain before DAS systems can complement or even replace BBOBS on a global scale. Foremost is the axial (single-component) directional sensitivity of DAS. Though work with helically wound optical fibers offering multi-component DAS sensitivity is underway^61^, modern BBOBS already provide four-component (three-component + pressure) recording capability with the same state-of-the-art instruments used in terrestrial networks. We noted that teleseismic waveforms recovered from the BDASA did not exhibit coherent strain amplitude when compared with particle velocity at BOST, suggesting that the mechanics of strain transfer from the solid earth across the cable housing and into the optical fiber are complex and deserve further study^62^. In the laboratory, DAS exhibits a linear frequency response, resulting in correct amplitude and distortion-free waves^24,28,63^, hence amplitude preservation may be currently limited by installation conditions and not by the DAS technology itself. Finally, ocean-bottom DAS deployments are not presently possible in remote oceanic locations. Most commercial DAS systems and laboratory measurements claim operation across up to 50 km of fiber, with sensitivity decreasing along the fiber due to optical attenuation. With the use of more complex pulse formats or distributed amplification, the sensing range can be extended to 70–100 km^64–66^ with a more even distribution of sensitivity along the fiber, while still using a standard telecom fiber installation. In principle, longer distances can be achieved with complex dedicated fiber installations and power supply along the fiber link (via use of optical repeaters^67,68^ and/or multiple stage distributed amplification^65,69^), but the impact on the cost and DAS sensitivity means that such systems are not currently practical.

## Methods

### Chirped-pulse DAS

A chirped-pulse DAS^29^ was used for the interrogator system, assisted by first-order co-propagating Raman amplification^66^. In comparison with conventional DAS systems, chirped-pulse DAS offers high signal-to-noise ratio (SNR) and low variations in sensitivity along the fiber^48,66,70^. The key of its performance lies in the use of a linearly chirped probe pulse for the time domain interrogation. Temperature or strain perturbations around the fiber affect its refractive index, which in turn slightly alters the central wavelength of the propagating light. An appropriately high linear chirp in the probe pulse (i.e., that inducing a spectral content much higher than the spectral content of the transform limited pulse) induces a local wavelength-to-time mapping arising from the temporal far-field condition^71^. Hence, variations in the central wavelength of the propagating light translate into temporal shifts in the trace at the particular location of the perturbation. The perturbation is then quantified by a time-delay estimation process via local trace-to-trace correlations over temporal windows similar to the probe pulse width.

The principle of operation of chirped-pulse DAS substantially improves the performance of the sensor over conventional DAS schemes. First, strain perturbations can be properly quantified by simply using direct detection. This contrasts with the conventional case, in which it is necessary to detect the trace optical phase for that purpose. Avoiding phase detection brings important advantages. Coherent detection imposes stringent requirement in the coherence length of the laser source, as it limits the DAS operation range due to the need for beating with a local oscillator. In chirped-pulse DAS, the coherence length of the probe laser can be relaxed, in principle simply requiring it to be substantially higher than the pulse width, with almost no detrimental effect on the acoustic SNR^72^. Polarization fading is not observed in chirped-pulse DAS (due to the use of direct detection). More importantly, sensitivity of conventional DAS completely fades in certain points along the fiber (acoustic SNR < 1 in up to 6% of fiber locations considering a healthy SNR optical trace) due to the impossibility of maintaining the phase reference in low intensity trace regions caused by its interferometric nature^73^. Those blind spots need to be corrected using complex post-processing techniques or multi-wavelength measurements^74^, typically at the expense of sensing bandwidth and higher measurements times. Chirped-pulse DAS, however, shows no fading sensitivity, enabling the raw strain signal as measured by the DAS to be directly processed without using any denoising/smoothing algorithm. This steady sensitivity is particularly beneficial for the subsequent 2D processing applied to isolate seismic events from other sources, since all points are captured with similar noise/sensitivity along the whole fiber length (>40 km)^70^.

In addition, signal attenuation due to fiber loss is greatly mitigated in our scheme with the use of distributed Raman amplification. Note that in Pastor-Graells et al.^66^, the fiber trace optical power fluctuation along a 75-km link is kept <7 dB, as opposed to the $\sim$28.5-dB attenuation expected without distributed amplification (28.5 dB $=$ 75 km $\times$ 2 $\times$ 0.19 dB, using 0.19 dB/km as typical standard single-mode fiber loss; note that roundtrip DAS attenuation is twice that of the fiber transmission losses). In this study, we observed DAS trace power fluctuations <3 dB along the 42-km fiber. This is in contrast with the optical signal attenuation of $\sim$16 dB ($={\!}42$ km $\times$ 2$\times$ 0.19 dB/km) expected without distributed amplification.

The optical resolution (or gauge length) and channel spacing of the employed sensor were both 10 m (equivalent to one seismometer placed every 10 m, measuring distributed strain over a length of 10 m), totaling 4192 channels over 42 km. Each channel was sampled at 1 kHz and later downsampled to 10 Hz in order to reduce the dataset size.

## Supplementary information

Supplementary Information

## Data Availability

Raw strain records from the BDASA are available on a public data repository at 10.22002/D1.1296. More information about reading and processing data files can be obtained from the authors upon request.

## Code availability

All codes required to reproduce the figures in this paper are written in Python and available from the authors upon request.
