# Supplementary material for: Distributed sensing of microseisms and teleseisms with submarine dark fibers
Source: Nat Commun. 2019 Dec 18;10:5778. doi: 10.1038/s41467-019-13262-7 (PMC6920360; doi:10.1038/s41467-019-13262-7)
Supplement: Supplementary file 1 — Supplementary Information [file 41467_2019_13262_MOESM1_ESM.pdf]

Supplementary Information

for

Distributed sensing of microseisms and teleseisms with submarine dark fibers

by Williams et al.

Supplementary Notes

Supplementary Note 1: Fiber-optic Cable

The BDASA occupied an optical fiber deployed within a power cable to the Belwind Offshore Wind Farm, offshore Belgium. The fiber is internally coupled with fillers to the cable's armor bedding (Supplementary Figure 1A). The cable consists of 3 core cables, an optical fiber, and a filler in polypropelene (PP) yarn. The outer serving in PP wraps the layer of round galvanized steel wires and is the layer that has direct contact with subsea sediments. Hence, vibrations that are passed from sediment into the fiber propagate through a frictional contact between adjacent components. The fiber and core cables are helically inserted into the cable.

Burial of the cable further attenuates vibrations generated by ocean gravity waves, as described in Godfrey, WO2018154275A1, 2018-02-09 [1]. This is clearly shown in Figure S1B, where the strength of observed ocean wave energy in the 0.01-0.2 Hz band decreases as a function of increasing burial depth. Figure S1B plots channels at constant water depth, as the change in ocean-bottom pressure associated with increasing water depth is a much stronger signal across the array.

Increasing depth of burial also attenuates temperature variations from the ocean water above. However, temperature variations within the cable due to changing electric load can exceed 1 K. We do not analyze the effect of temperature in-situ.

Supplementary Note 2: 0.18 Hz Model

In order to fit depth-dependence of noise at the primary microseism peak (0.18 Hz), we first calculate a theoretical curve for the pressure at the seafloor under an ocean surface gravity wave as a function of seafloor depth. Here, we consider only p_d, the dynamic pressure due to wave propagation. The pressure profile with water depth for ocean surface gravity waves over a flat bed is given as

$$p_d(t,x) = \rho g \eta(t,x) \frac{\cosh(k(H+z))}{\cosh(kH)}$$

where ρg is the specific weight of water, $\eta(t,x)$ represents the sea-surface height along the propagating surface gravity wave, H is depth to the seafloor, and k is angular wavenumber [2]. Evaluated at the seafloor ($z=-H$), we find: $p_d(x) \propto \mathrm{sech}(k(x)\, H(x))$. In order to evaluate this expression, we solve the implicit dispersion relation for surface gravity waves ($\omega^2 = gk\tanh(kH)$) to find angular wavenumber $k = k(x)$ using an iterative scheme given the depth profile of the seabed $H(x)$ and frequency $\frac{\omega}{2\pi} = 0.18$ Hz. Finally, we perform a linear regression to find a single constant of proportionality between the Fourier

amplitude at 0.18 Hz and our theoretical $p_d(x)$ as a function of depth/distance (i.e. $FFT_t\{\varepsilon\}(f = 0.18\text{Hz}, x) = A\,p_d(x) + B$). The resulting pressure-depth model is plotted against BDASA data in Fig. 3. We only perform this fit further than 12 km from shore where water depth is > 10 m, as shoaling waves in shallow water do not adhere to linear wave theory. We neglect any effects of variable burial depth of the fiber.

Supplementary Note 3: Directional Spectra

The directional spectra plotted in Fig. 7 (polar diagrams) are calculated from the frequency-wavenumber spectrum of raw BDASA strain records. For each wave type, we first assume a dispersion relation $\omega = \omega(k)$ and then evaluate ω for a range of apparent wavenumbers $k_a = k/\cos(\theta)$, corresponding to waves propagating across the array from oblique azimuths. For ocean surface gravity waves (Fig. 7A), we use the relation $\omega^2 = gk\tanh(kH)$. For Scholte waves (Fig. 7B), we use only 1.5-3.5 Hz, where the observed f-k spectrum appears non-dispersive, and assume constant phase velocity ($\omega \propto k$). The mean f-k amplitude is then obtained for each incident azimuth θ by interpolating the f-k spectral amplitudes along each calculated dispersion curve and averaging them. To separate the incoming and outgoing energy, we perform this calculation independently for f-k quadrants 1 and 2. We plot only $0 - 180^o$ because quadrants 1 and 3 (similarly, 2 and 4) are symmetrical by nature of the 2D FFT, so we cannot distinguish the direction of energy propagating perpendicular to the array (whether SW-NE or NE-SW).

Supplementary Note 4: Teleseism Extraction by Filtering

The superposition of coherent signals from ocean waves, Scholte waves, and teleseism in BDASA data makes interpretation of raw strain records challenging (Fig. 2A). Because these signals also inhabit overlapping frequency bands, simple time-domain or time-frequency filtering is insufficient to isolate individual signals. Instead, we employ a frequency-wavenumber filtering approach that exploits the dense spatial sampling and wide aperture of the BDASA. We first apply a 2D Hamming (cosine-sum) taper $W_H[n, m]$ to the raw t-x domain strain data $\varepsilon(t, x)$ and then compute the 2D Fast Fourier Transform (FFT) to obtain the f-k spectrum $\hat{\varepsilon}(f, k)$.

$$W_H[n, m] = \left(\frac{25}{46} - \frac{21}{46}\cos\left(\frac{2\pi n}{N_t}\right)\right)\left(\frac{25}{46} - \frac{21}{46}\cos\left(\frac{2\pi m}{N_x}\right)\right)$$

$$\hat{\varepsilon}(f, k) = FFT_{2D}\{W_H\,\varepsilon(t, x)\}(f, k)$$

In the f-k domain, the spectrum is organized according to apparent phase velocity along the array. We only transform data from quasi-linear array segments because this simplifies interpretation of the f-k spectrum relative to a single reference direction (the axis of the fiber). Teleseismic phases from the Fiji deep earthquake, which is nearly antipodal to the BDASA, arrive with apparent horizontal velocity > 10 km/s, and for non-dispersive body waves the energy should appear in the f-k domain along a line of constant f/k. However, the aperture of the BDASA determines wavenumber domain sampling, relegating energy from teleseismic phases to the zero-wavenumber bin across most of the frequency range of interest. For example, a 5-km transformed segment with 500 channels at 10-m spacing has 0.0002 m^{-1}-wide wavenumber bins, and the wavenumber of a 1 Hz teleseismic P-wave arriving at apparent horizontal velocity $c = f/k \approx 10000$ m/s is ~ 0.0001 m^{-1}. The f-k domain also contains directional information: for the BDASA, energy that appears in f-k quadrants 1 and 3 corresponds to waves propagating land-ward (from the north/west) across the array, and energy that appears in quadrants 2 and 4 corresponds to waves propagating sea-ward (from the south/east). Hence, teleseismic phases from the Fiji earthquake only appear in f-k quadrants 1 and 3.

In conventional f-k processing, we would apply a dip filter to isolate a non-dispersive signal, which passes a sector between two lines of constant f/k. However, we found that this approach is not numerically stable for low frequencies where the pass sector is only a few bins wide. Consequently we reverted to a simple approach, using a 2D rectangular bandpass filter between 0.001-1 Hz and 0-0.002 m^{-1}, without any tapering (Supplementary Figure 2). We apply this filter only in f-k quadrants 1 and 3 to attenuate all energy propagating across the BDASA from the south/east. With $f_1 = 0.001\text{Hz}$, $f_2 = 1\text{Hz}$, $k_1 = 0\text{m}^{-1}$, $k_2 = 0.002\text{m}^{-1}$, $f_m = (f_1 + f_2)/2$, and $k_m = (k_1 + k_2)/2$, the filter $H(f, k)$ can be expressed as:

$$H(f,k) = \Pi\left(\frac{f - f_m}{f_2 - f_1}\right)\Pi\left(\frac{k - k_m}{k_2 - k_1}\right) + \Pi\left(\frac{f + f_m}{f_2 - f_1}\right)\Pi\left(\frac{k + k_m}{k_2 - k_1}\right)$$

where Π denotes a rectangular boxcar of unit amplitude. This filter is non-causal ($h(t, x) = FFT_{2D}^{-1}\{H(f, k)\}$ is even) and exhibits some Gibbs ringing because of its finite implementation, so a more careful approach may need to be considered for accurate seismic travel-time picking in future studies. After filtering, some residual energy from Scholte waves remains, so we stack across a 5-km segment to improve SNR and isolate teleseismic body waves. When the stack is applied across many sub-sections of the array, relatively high spatial coherence is observed for both P- and S-wave phases (Supplementary Figure 5).

Supplementary Note 5: Teleseism Waveform Fidelity

As discussed in the main text, recovered P-waves exhibit low-to-moderate waveform fidelity at high frequencies and recovered S-waves exhibit moderate-to-high waveform fidelity at low frequencies. Fig.

S3A shows the evolution of P waveforms along the array, showing that some coherent energy arrives before the first arrival because of our acausal filter. Overall, P-wave fidelity is low, with a maximum correlation coefficient of 0.26 in the 0.5-1 Hz frequency band calculated in a window centered between the PKP and pPKP arrivals (Supplementary Figure 3). However, a high spike in correlation coefficient up to 0.39 is observed when the first PKP motions enter the correlation window (Supplementary Figure 3B), suggesting that the BDASA beam trace contains sufficient phase information at high frequencies to permit correlation-based detection algorithms such as template matching. Overall, S-wave fidelity is moderate to high, with a maximum correlation coefficient of 0.60 in a window centered around the SS phase, and average correlation coefficient greater than 0.40 throughout the complete S-wave train (Supplementary Figure 4).

Supplementary Note 6: Teleseism Amplitude Comparison

The 2018-08-19 $M_w 8.2$ Fiji deep earthquake is an atypical event to consider when testing the seismic monitoring capabilities of an instrument, so we include some comparative analysis here. With an epicentral depth around 600 km, the Fiji earthquake did not produce a significant surface wave train. The BDASA was also recording at an epicentral distance of 146°, in the "shadow zone," meaning that the primary body phases observed were PKP and SS, the former of which can be strongly attenuated. Comparing the velocity spectrum of the Fiji earthquake recorded at BOST with mean velocity spectra of regional and teleseismic earthquakes [3], we observe an expected correspondence between the Fiji event and mean $M8.0$ event over a broad band, with stronger S-wave energy at low frequencies than in the mean $M8.0$ event (Supplementary Figure 6). Because we have recovered the principal phases of the Fiji earthquake between 0.01-1 Hz on the BDASA, even in a high-noise shallow-water environment, we can assume that the spectrum observed on nearby broadband BOST exceeds the instrumental noise floor of the BDASA across this band. Hence, we can compare the mean spectra of other event sizes and distances from clinton:2002 indirectly with our demonstrated detection capabilities. As shown in Supplementary Figure 6, the Fiji earthquake observed at BOST and BDASA is a relatively weak signal, with regional earthquakes ($\sim$ 100 km epicentral distance) above $M3.5$ exceeding this threshold across most of their band.

References

1. Godfrey, A. *Monitoring subsea cables* WO2018154275A1, 2018-02-09. `https://patents.google.com/patent/WO2018154275A1/en`.

2. Holthuijsen, L. *Waves in Oceanic and Coastal Waters* (Cambridge University Press, 2007).

3. Clinton, J. & Heaton, T. Potential Advantages of a Strong-motion Velocity Meter over a Strong-motion Accelerometer. *Seismological Research Letters* **73,** 332–342 (2002).

Supplementary Figures

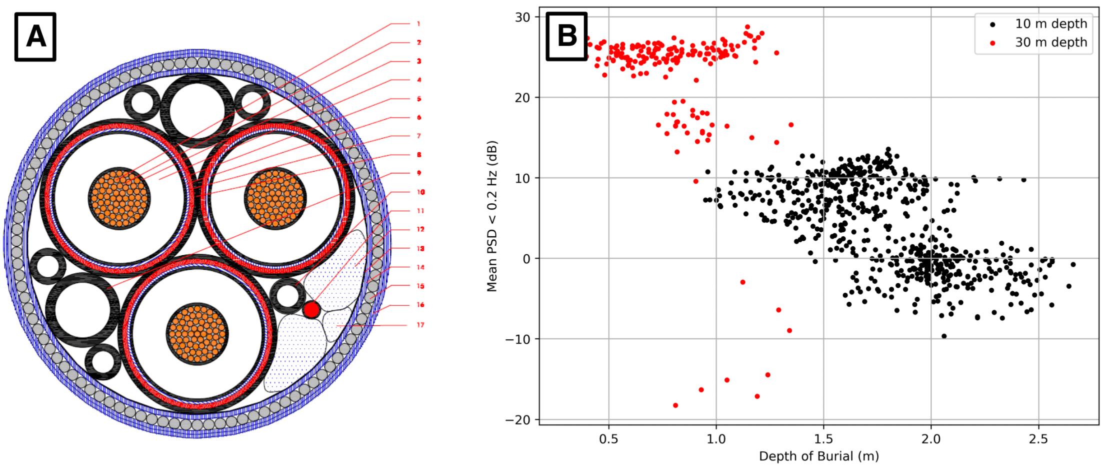

Supplementary Figure 1: Cable coupling (A) Schematic cable cross-section. Number 11 (red) indicates the position of the fiber. (B) Scaling of observed ocean wave energy with depth of burial for each of two water depths (10 m in black, 30 m in red).

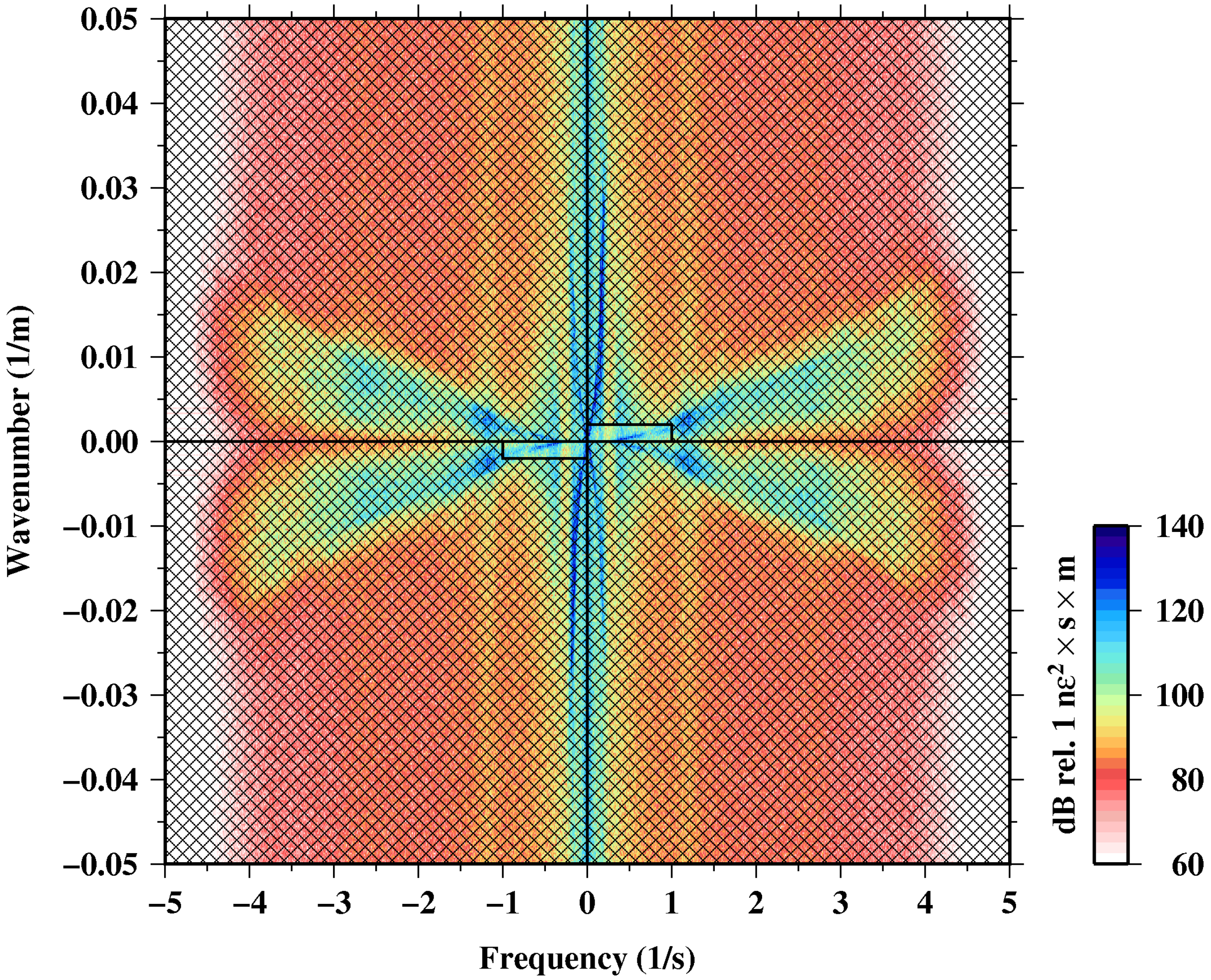

Supplementary Figure 2: Frequency-wavenumber filter The rectangular frequency-wavenumber filter applied to preserve only seismic waves in quadrants 1 and 3, indicating propagation from the north/west. Shaded regions are zero, unshaded regions are 1. The inverse 2D Fast Fourier Transform was computed, time-series from each channel between 35 and 40 km were stacked, and finally a bandpass filter was applied to produce the waveforms shown in Fig. 9, Supp. Fig. 3, and Supp. Fig. 4.

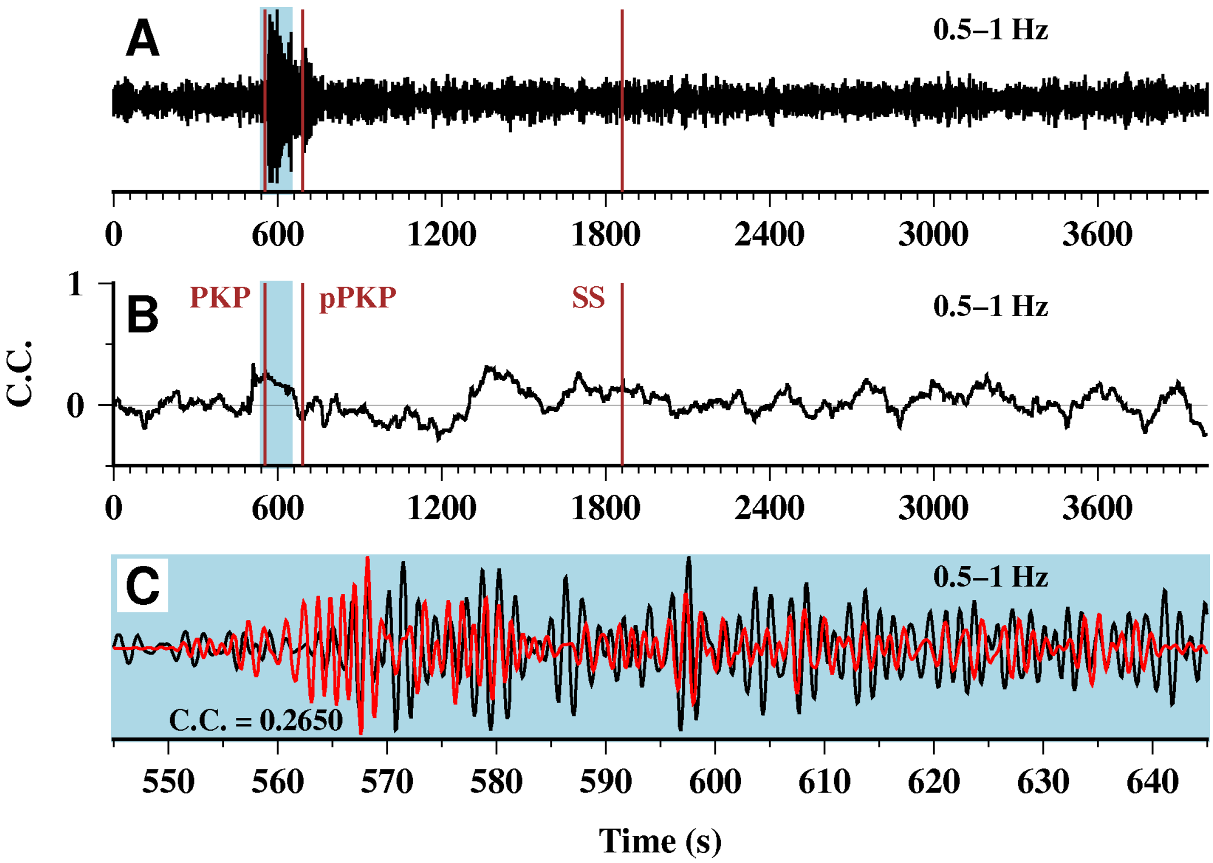

Supplementary Figure 3: P-wave fidelity (A) BDASA beam trace filtered 0.5-1 Hz (same as shown in Fig. 9c). (B) Correlation coefficient (C.C.) between the DAS and BOST waveforms filtered 0.5-1 Hz calculated over a 120 s moving window. (C) Blow-up of 1860-2320 s for the waveforms filtered 0.5-1 Hz around the arrival of the PKP phase just after 550 s, showing low-to-moderate waveform coherence between BOST (red) and BDASA (black) (C.C. = 0.2650). The time-shift between BOST and BDASA ($\sim$ 50 km apart) has not been removed.

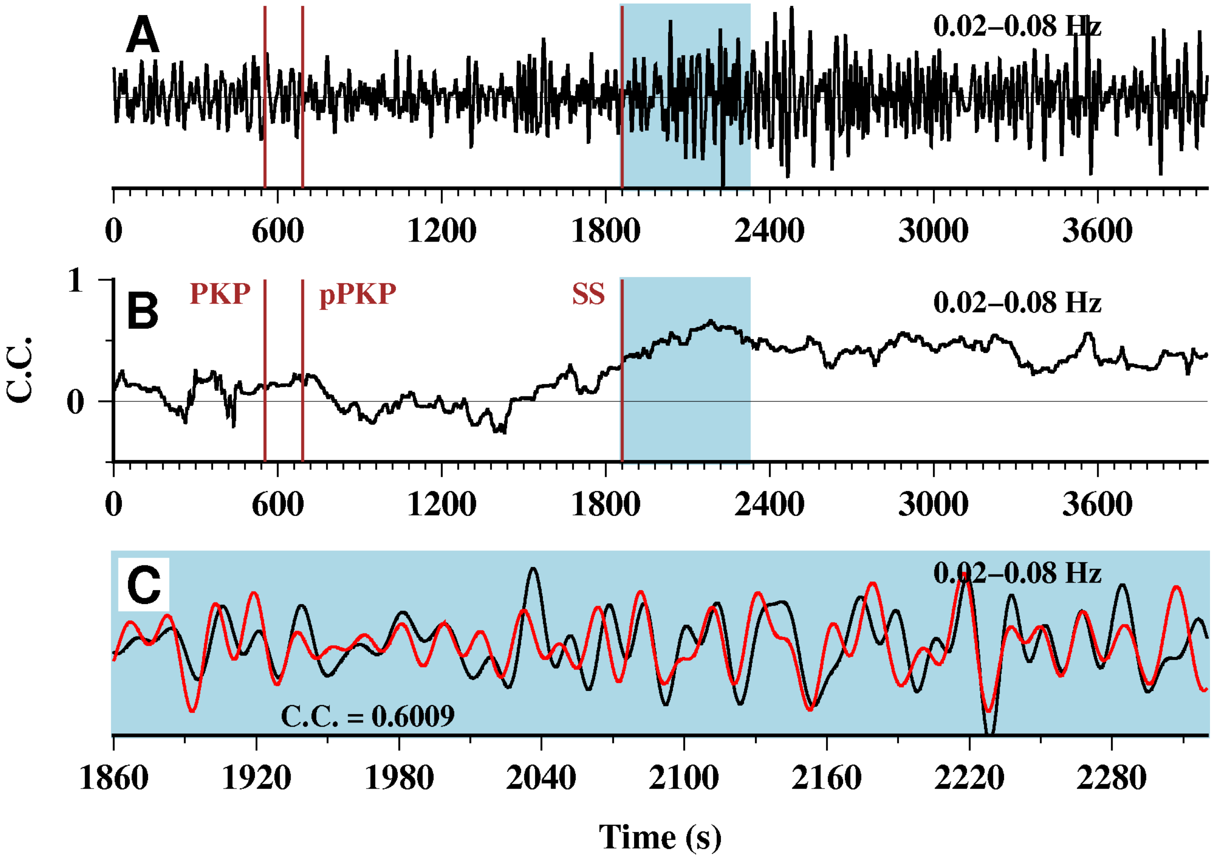

Supplementary Figure 4: S-wave fidelity (A) BDASA beam trace filtered 0.02-0.08 Hz (same as shown in Fig. 9c). (B) Correlation coefficient (C.C.) between the DAS and BOST waveforms filtered 0.02-0.08 Hz calculated over a 240 s moving window. (C) Blow-up of 1860-2320 s for the waveforms filtered 0.02-0.08 Hz around the arrival of the SS phase just after 1860 s, showing moderate-to-high waveform coherence between BOST (red) and BDASA (black) (C.C. = 0.6009).

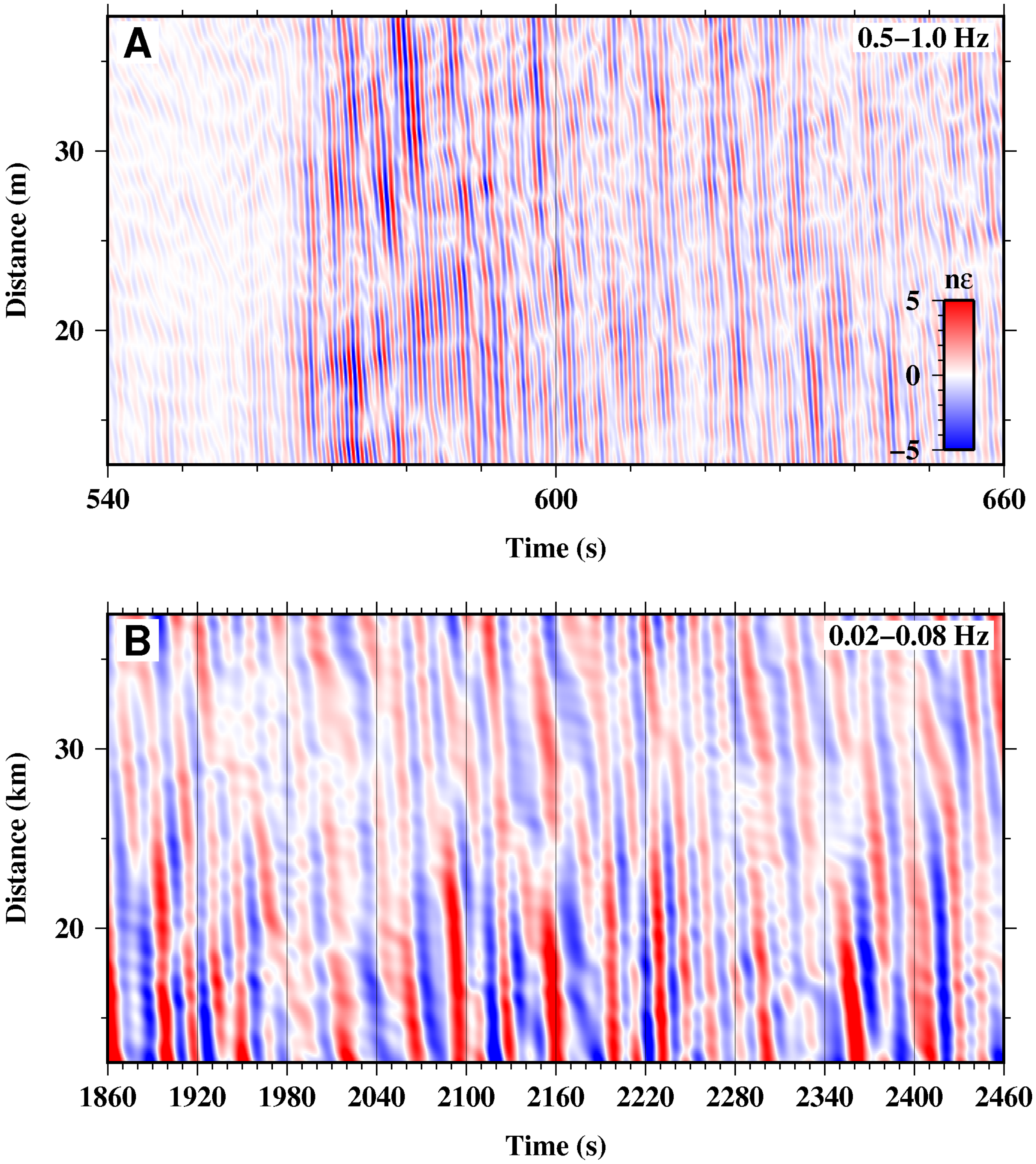

Supplementary Figure 5: Waveform coherence F-k filtered waveforms as shown in Figure 9 stacked in a 5-km moving window between 10 and 40 km (plotted at the midpoint of the stacked interval). (A) Bandpassed 0.5-1 Hz, showing the arrival of the PKP and pPKP phases, and (B) bandpassed 0.02-0.08 Hz, showing the arrival of the S-wave train. Note that the filtering procedure applied is non-causal, so some coherent PKP energy can be observed before the true PKP arrival, especially between 12-20 km. A similarly effective causal filter could be designed for more accurate travel-time picking.

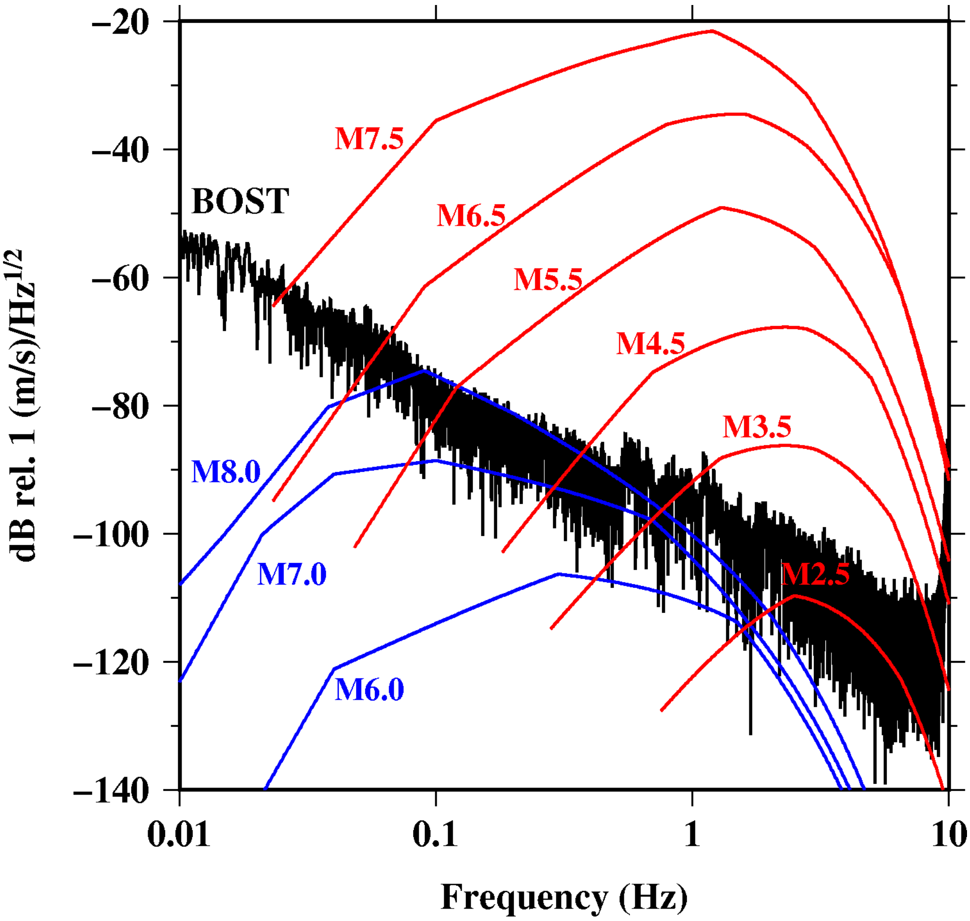

Supplementary Figure 6: Earthquake scaling BOST.BHE spectrum of the 2018-08-19 $M8.2$ Fiji deep earthquake (black) compared with average spectra of teleseismic (blue, $\sim$ 3000 km) and regional (red, $\sim$ 100 km) earthquakes from [3] (converted from acceleration into velocity units).
